# An Update on the Metabolic Roles of Carbonic Anhydrases in the Model Alga *Chlamydomonas reinhardtii*

**DOI:** 10.3390/metabo8010022

**Published:** 2018-03-13

**Authors:** Ashok Aspatwar, Susanna Haapanen, Seppo Parkkila

**Affiliations:** 1 Faculty of Medicine and Life Sciences, University of Tampere, FI-33014 Tampere, Finland; Haapanen.Susanna.E@student.uta.fi (S.H.); seppo.parkkila@staff.uta.fi (S.P.); 2 Fimlab, Ltd., and Tampere University Hospital, FI-33520 Tampere, Finland

**Keywords:** carbonic anhydrases, CA gene family, *Chlamydomonas reinhardtii*, model alga, metabolic role, photosynthesis

## Abstract

Carbonic anhydrases (CAs) are metalloenzymes that are omnipresent in nature. CAs catalyze the basic reaction of the reversible hydration of CO_2_ to HCO_3_^−^ and H^+^ in all living organisms. Photosynthetic organisms contain six evolutionarily different classes of CAs, which are namely: α-CAs, β-CAs, γ-CAs, δ-CAs, ζ-CAs, and θ-CAs. Many of the photosynthetic organisms contain multiple isoforms of each CA family. The model alga *Chlamydomonas reinhardtii* contains 15 CAs belonging to three different CA gene families. Of these 15 CAs, three belong to the α-CA gene family; nine belong to the β-CA gene family; and three belong to the γ-CA gene family. The multiple copies of the CAs in each gene family may be due to gene duplications within the particular CA gene family. The CAs of *Chlamydomonas reinhardtii* are localized in different subcellular compartments of this unicellular alga. The presence of a large number of CAs and their diverse subcellular localization within a single cell suggests the importance of these enzymes in the metabolic and biochemical roles they perform in this unicellular alga. In the present review, we update the information on the molecular biology of all 15 CAs and their metabolic and biochemical roles in *Chlamydomonas reinhardtii*. We also present a hypothetical model showing the known functions of CAs and predicting the functions of CAs for which precise metabolic roles are yet to be discovered.

## 1. Introduction

Carbonic anhydrases (EC 4.2.1.1) (CAs) are metalloenzymes that catalyze the reversible reaction of hydration of carbon dioxide to bicarbonate (CO_2_ + H_2_O ⇔ HCO_3_^−^ + H^+^). The CAs belong to seven evolutionarily unrelated CA-gene families (α-, β-, γ-, δ-, ζ-, η-, and θ-CAs) [1,2,3,4,5].

The CAs are widespread in nature, and are found abundantly in plants, animals, and microorganisms, suggesting that the CAs have many diverse metabolic roles in living organisms [6,7,8]. Vertebrates and mammals have only α-CAs and contain multiple isoforms of the enzyme. In contrast, multicellular plants and unicellular photosynthetic organisms seem to have members of six CA gene families, often multiple isoforms of CAs from each gene family [4,9]. The *Chlamydomonas reinhardtii* genome analysis has revealed the presence of at least 15 CA genes encoding three different families of CAs. The number of CAs in *C. reinhardtii* is thus much higher than previously thought for a unicellular cell alga. Interestingly, a recent study showed that the limiting CO_2_-inducible B protein (LCIB) family belongs to the β-CAs [10]. The amino acid sequences of these CA families are different, but most of these CA families have a Zn^2+^ atom at the active site [11]. In this alga, CAs have been found in the mitochondria, chloroplast thylakoid, cytoplasm, and periplasmic space [12,13]. A recent study showed that CAH6 is localized in the flagella instead of the pyrenoid stroma, as previously reported [14].

The downregulation of CA activity using molecular techniques and chemical inhibitors has shown reduced lipid biosynthesis in chloroplasts compared with chloroplasts from wild-type plants [15]. CAs are indirectly involved in lipid synthesis (and perhaps other HCO_3_^−^-requiring pathways in plastids), serving to “concentrate” CO_2_ in plastids as HCO_3_^−^, and reduce the rate of CO_2_ diffusion out of plastids [15]. The CA might indirectly influence fatty acid synthesis in plastids by modulating plastidial pH, as the enzyme fatty acid synthase activity requires an optimal pH for fatty acid synthesis [15].

The role of CAs in pH regulation is well known in animal cells. However, the roles of CAs in pH regulation in this model alga are not known, and need to be investigated. The presence of 15 CAs in *C. reinhardtii* suggests that they are involved in several other metabolic functions in addition to a CO_2_-concentrating mechanism (CCM), which is attributed to the evolutionarily conserved enzymes in plants. In *C. reinhardtii*, CAs are involved in many metabolic functions that involve carboxylation or decarboxylation reactions, including both photosynthesis and respiration. In addition, it has been clearly shown that CAs also participate in the transport of inorganic carbon to actively photosynthesizing cells and away from nonphotosynthesizing, respiratory cells [12,16].

In the current article, we will review the information on CAs of *C. reinhardtii*, a unicellular model alga. We will describe the information that is available on the molecular biology, and present the data for the metabolic and biochemical roles of the three CA gene families. For each CA enzyme from the three CA families, we will highlight the current research and questions that have been addressed by researchers in the field. We will also present a hypothetical model showing the known functions of CAs and predicting the functions of CAs for which precise metabolic roles are yet to be discovered. Finally, we present future directions in the field of *C. reinhardtii* CA research in order to study the precise metabolic and physiological roles of CAs from this alga.

## 2. General Aspects of Carbonic Anhydrases

Carbon dioxide (CO_2_) is a very important molecule that is found in all living organisms. CO_2_ is soluble in lipid membranes, and freely diffusible in and out of the cell [17,18,19]. Carbon dioxide and bicarbonate constitute the main buffer system for pH regulation in all living cells. CAs play a very important role in the transport of CO_2_ and protons across cell membranes [20,21]. CA families differ in their preference for the metal ions used within the active site for performing the catalysis. The general enzyme catalytic mechanism of all of the CAs involves a reaction between a metal cofactor bound to OH^−^ and CO_2_, giving rise to a HCO_3_^−^ ion that is subsequently replaced from the metal with an H_2_O molecule. This reaction is shown in Equation (1) below, where Enz indicates CA enzymes, and M indicates the metal cofactor. The regeneration of OH^−^ involves a transfer of H^+^ from the metal bound to the H_2_O molecule to the solvent, as shown in Equation (2) [22,23].

(1) $$\mathrm{EnzM}-\mathrm{OH}+{\mathrm{CO}}_{2}\Leftrightarrow \mathrm{EnzM}-{\mathrm{HCO}}_{3}{}^{-}\overset{H_{2}O}{\Leftrightarrow }\mathrm{EnzM}-H_{2}O+{\mathrm{HCO}}_{3}{}^{-}$$

(2) $$\mathrm{EnzM}-H_{2}O\Leftrightarrow \mathrm{EnzM}–{\mathrm{OH}}^{-}+H^{+}$$

CAs are metal-containing enzymes that are found in every living organism and have been studied extensively in the past. These enzymes are widely distributed among metabolically diverse species from all three domains of life. CAs perform various physiological functions, such as respiration, photosynthesis, pH regulation, ion transport, bone resorption, and the secretion of gastric juice, cerebrospinal fluid, and pancreatic juice [8,24,25]. CAs are also involved in electrolyte secretion, CO_2_ and pH homeostasis, CO_2_ fixation, and biosynthetic reactions, such as gluconeogenesis and ureagenesis [26,27,28,29].

**Figure 1:**
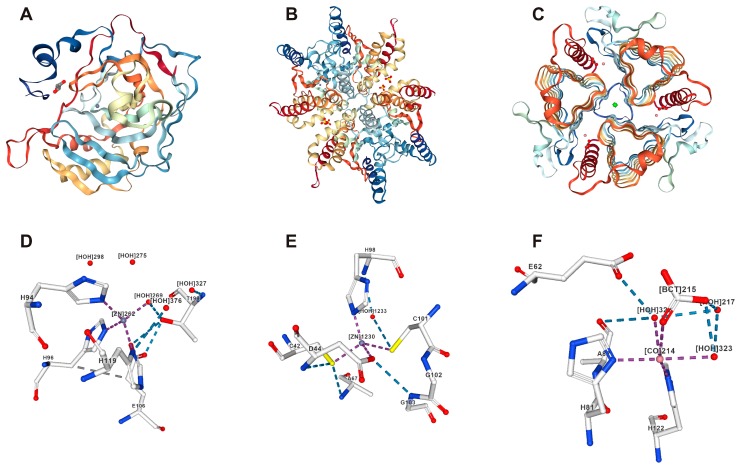
Representative structures of the α-CA, β-CA, and γ-CA families of enzymes and their ligand-binding sites. (**A**) Structure of human CAII enzymes retrieved from PDB 3U45. The human CAII monomer mostly consists of beta strands and contains a single active site with three zinc-coordinating histidine residues [38,39]; (**B**) Structure of *Haemophilus influenzae* β-CA retrieved from PDB 2A8C [40]; (**C**) Structure of γ-CA from *Methanosarcina thermophila* 1QRE [41,42]; (**D**–**F**) Metal at the active site coordinated with histidine residues (purple), hydrogen bonds (blue), halogen bonds (turquoise), hydrophobic contacts (gray), and pi interactions (orange, green). Images D and E show Zn^2+^ at the active site, and image F shows the Co^2+^ substitution in the structure.

Although the CA families are diverse and widespread, the current understanding of their biological role is mainly based on studies with several α and β-class CAs. Among the other CA classes, the δ and ζ classes have been reported only in diatoms and coccoliths [30,31]. Cam is the γ-class archetype isolated from *Methanosarcina thermophila*, an anaerobic methane-producing species from the Archaea domain [9]. The η and θ-class CAs have been identified recently from *Plasmodium falciparum* and marine diatoms, respectively [1,4]. The α-CAs are typically found as monomers and dimers; the β-CAs are typically found in many oligomerization states; and the γ-CAs are typically found as trimeric forms. Table 1 shows details of all of the CA gene families of enzymes, and Figure 1 depicts example structures of the major CA families: α, β, and γ.

The α-carbonic anhydrases: Among the CA gene families, the α-CA was first to be discovered in 1932 in erythrocytes [43,44]. Among the CA gene families, α-CAs are the most widely distributed CA family. α-CAs are also the most studied CA-group, probably because all of the human CAs belong to this enzyme family. In humans, there are 15 α-CA family isoforms; among these, 12 α-CA isoforms are catalytically active, and three CAs do not have catalytic activity, and are designated carbonic anhydrase-related proteins (CARPs) [45]. The α-CAs are monomers that are found in every type of tissue; furthermore, these enzymes have unique localizations within the cell. These enzymes have Zn^2+^ as a metal ion at their active site, and are coordinated by three histidine residues. The α-CAs play a wide variety of roles in living organisms.

The β-carbonic anhydrases: The first β-CA was discovered in 1938, and subsequently, many β-CAs have been reported from bacteria, archaea, yeasts, algae, and plants [32,46,47]. The β-CAs contain Zn^2+^ as the metal ion at the active site of the enzyme. In β-CAs, the Zn^2+^ atom is coordinated by two cysteines and one histidine, unlike the three histidines found in α and γ-CAs [48]. Some of the enzymes in the β-CA class have four zinc ligands, that is, one His residue, two Cys residues, and one Asp residue coordinated to Zn^2+^ [49]. β-CAs are found in many oligomerization states due to the presence of an α/β-fold that promotes an association with the formation of dimers. Several crystal structures of β-CAs have already been reported [50,51,52]. The β-CAs play important roles in CO_2_ fixation in plants and other photosynthetic organisms, as well as in pH regulation, survival, virulence, and invasion in bacteria [53,54,55].

The γ-carbonic anhydrases: The γ-CAs were discovered in 1994, and are found in *Archaea, Bacteria,* and plants [56,57]. These enzymes are trimers, unlike the other CA gene families. The γ-CAs contain Zn^2+^ at the active site, and in some cases, the enzymes contain Fe^2+^ instead of the Zn^2+^ that is found in anaerobic *Archaea* [58]. The active site metal is coordinated by three histidine ligands and one water molecule [42]. The kinetic studies of γ-CA from *M. thermophila* showed that the hydration of CO_2_ is a two-step process similar to that in α-CAs [59,60].

The δ-carbonic anhydrases: The first δ-CA was characterized from *Thalassiosira weissflogii*, a unicellular microalga representing a species of centric diatoms [61]. The δ-CAs are also present in many eukaryotic marine phytoplanktons [8,61]. The crystal structure of δ-CAs has not been resolved, but studies using X-ray absorption spectroscopy methods have shown that the metal ion Zn is coordinated by histidine ligands similar to those in the α and γ classes of CAs [48].

The ζ-carbonic anhydrases: Xu et al. described the ζ-CAs in marine diatoms in 2008 [8]. The ζ-CAs contain cadmium at the active site of the enzyme as an alternative metal cofactor [33]. The structure of CdCA1 has been resolved, and it is reported that the metal-binding region is repeated three times in this enzyme [62]. The ζ-CAs have been shown to bind to other metals apart from Cd and remain active. The reason for having Cd at their active site is possibly adaptation in the ocean, where Zn levels are often low.

The η-carbonic anhydrases: η-CA is a novel class of CA that Del Prete et al. recently discovered recently [1]. The η-CA is found in the malaria-causing protozoan parasite *Plasmodium falciparum*. The active site of η-CA contains Zn^2+^, and is coordinated by two Hi residues and one Gln residue, in addition to the water molecule/hydroxide ion acting as a nucleophile in the catalytic cycle [37]. This arrangement is unique compared with that in the other CA gene families [37].

The θ-carbonic anhydrases: The θ-CA family is a recent addition to the existing CA gene family, as reported by Kikutani et al. [4] in 2016. These CAs have also been described in a cyanobacterium by Jin et al. [10]. The θ-CAs are implicated in the formation of CO_2_ from HCO_3_^−^ in the chloroplast.

### 2.1. Carbonic Anhydrases in Photosynthetic Organisms

Photosynthetic organisms contain CAs that belong to six different CA gene families, namely: α-CAs, β-CAs, γ-CAs, δ-CAs, ζ-CAs, and θ-CAs. Each of the at least three gene families of α-CAs, β-CAs, and γ-CAs are represented by multiple isoforms in all of the species. The γ-CAs are also found in photosynthetic bacteria [63,64]. θ-CA has been recently discovered in the thylakoid lumen of the marine diatom *Phaeodactylum tricornutum* [4]. The four CA gene families (α-, β-, γ-, and θ-CAs) that are found in photosynthetic organisms contain zinc as a metal ion at the active site of the enzymes [4,8]. Due to the alternative splicing of CA transcripts, the number of functional CA isoforms in many of the species is greater than the number of genes that encode a particular CA enzyme. In photosynthetic organisms, CAs are expressed in different cellular compartments, and are most prevalent in chloroplasts, cytosol, and mitochondria. The diversity in location suggests the importance of the CAs in the many physiological and biochemical roles they may play in photosynthetic organisms.

### 2.2. Carbonic Anhydrases in Chlamydomonas Reinhardtii

The model alga *Chlamydomonas reinhardtii* is a unicellular photosynthetic eukaryote that contains multiple genes encoding CAs for three different gene families. The α-CAs were discovered in the 1980s and 1990s in *C. reinhardtii* [12,65,66]. The β-CAs were discovered during the 1990s, [67,68,69], and with the sequencing of the complete genome of *C. reinhardtii*, three novel γ-CAs were found in the latter part of the 2000s [57,70,71,72].

The alga *C. reinhardtii* has three α-CAs, nine β-CAs (including the recently discovered three homologs of the LCIB protein family), and three γ-CAs [10]. Among the CAs that are found in *C. reinhardtii,* the β-CAs are predominant, with the highest isozyme number in this organism. Details of all the CAs that have been discovered in *C. reinhardtii* to date are presented below (Table 2).

#### 2.1.1. α-Carbonic Anhydrase 1

Among the CA genes, *α-Ca1* was the first gene that was identified in *C. reinhardtii* in the 1980s [65,66,94]; it was named *Ca1*, reflecting the order of discovery. Several groups have shown that CAH1 is localized in the periplasmic space of the alga [65,66,94]. *Cah1*, the gene encoding CAH1, has been cloned [78]. The cDNA encodes a polypeptide of 377 amino acid residues. It is composed of a signal peptide that is 20 amino acids long, a small subunit, a large subunit, and a spacer region between the subunits [76,78]. Fujiwara et al. [66] discovered that the gene sequence is 93.6% identical to the sequence of *Cah2*, which encodes CAH2. In addition, their intron insertion sites are identical. These findings indicate that *Cah1* and *Cah2* are paralogs or the products of gene duplication [66].

The expression of CAH1 can be induced in the presence of low amounts of CO_2_ compared to high amounts of CO_2_. However, Kucho et al. [75] showed that in addition to low amounts of CO_2_, CAH1 requires the presence of light for its induction [75]. Additional studies have shown an accumulation of CAH1 when the CO_2_ concentration is reduced in the presence of light [65,66]. Inhibition of the photosynthetic reaction using 3-(3,4-dichlorophenyl)-1,1-dimethylurea (DCMU) leads to a reduction in CAH1 mRNA, suggesting that the accumulation of CAH1 mRNA requires functioning photosynthesis [65]. CO_2_ regulates the induction of CAH1 through various enhancer and silencer sites [75]. At least a 692-bp region from -651 to +41 relative to the transcription start site was detected to be adequate for the full induction of CAH1 in response to light and low CO_2_ [75]. Kucho et al. [75] identified a crucial regulatory area (63 bp from −293 to −231 relative to the transcription start site) that contains two enhancer elements. In addition, they detected DNA-binding proteins that specifically interact with these enhancer elements in the presence of light and low CO_2_ conditions [74]. Additionally, other silencers and enhancers have been found, but they are usually responsible for only small changes in the induction or downregulation of CAH1 [75].

The physiological role of CAH1 has already been extensively discussed in the earlier review [12]. CAH1 provides more C_i_ to the *C. reinhardtii* cell in a C_i_-deficient environment [12]. Nonetheless, many studies have shown that CAH1 mutant cells are as viable as the wild type under the conditions assayed. In contrast, drug inhibition restricts the growth, which indicates that other CAs, such as CAH2 and CAH8, might maintain the necessary CA activity in CAH1-deficient cells [12].

#### 2.1.2. α-Carbonic Anhydrase 2

CAH2 was discovered at same time as CAH1 by Fukuzawa et al. [65,80,81]. CAH2 is a periplasmic protein and is a heterotetramer, as is CAH1. CAH2 consists of two identical large subunits and two small subunits [66]. The molecular weight of the holoenzyme is approximately 84.5–87.9 kDa. The large subunit is 38 kDa in size, and the small one is 4.2 kDa, which are result of the cleavage of the subunits of the proprotein. Therefore, the subunits are comparatively larger than the corresponding units in CAH1 [80]. The genetic similarity has already been stated, but the similarity of the amino acid sequences is 91.8% [66]. Nevertheless, the catalytic activity of CAH2 is approximately 1.6 times that of CAH1, as that of CAH2 is 3300 units per mg protein compared to 2200 units per mg protein with CAH1 [80]. The subunits of CAH2 are bound to each other with disulfide bonds, as in CAH1. CAH2 also has similar glycosylation sites to those of CAH1 in the large subunit [80].

The expression of CAH2 is more abundant than that of CAH1, and the expression of CAH2 is greatly induced in low CO_2_ conditions as opposed to CAH1, which is moderate in amount and is present in high CO_2_ conditions [66]. Furthermore, Tachiki et al. [80] have suggested that CAH2 might be present in low as well as high CO_2_ conditions, as *Cah2* mRNA is expressed in both conditions [80]. The function and role has been suggested to be the same as that of CAH1, and Rawat et al. [81] proposed that *Cah2* could represent a gene duplication, without a specific role of its own [81].

#### 2.1.3. α-Carbonic Anhydrase 3

Among the α-CAs of *C. reinhardtii,* Karlsson et al. identified α-CAH3 in the late 1990s [82], and showed that it was localized in the thylakoid lumen [82,84,91]. CAH3 is a 29.5-kDa polypeptide that Karlsson et al. originally isolated in 1995 [84]. The longest cDNA clone obtained from the cDNA library consisted of 1383 bp and contained an open reading frame that encoded a polypeptide of 310 amino acids [82].

CAH3 functions in the thylakoid lumen, and has been suggested to be part of photosystem II (PSII) or CCM [13,88,95,96]. Hanson et al. [13] showed that cia3, which is a mutant line of *C. reinhardtii* lacking functioning CAH3, has a limiting effect on the function of Rubisco in vivo [13]. The physiological function of CAH3 is also related to the location within thylakoids; thus, in stromal thylakoids, CAH3 is probably associated with light reactions of photosynthesis, and in the intrapyrenoid thylakoids, CAH3 is presumably connected to the actions of Rubisco [88].

In addition, the actions of CAH3 are connected to the fatty acid composition of the thylakoid membranes [88]. In low CO_2_ conditions, the activity of CAH3 is implicitly related to an increase in the relative amount of polyunsaturated fatty acids. The change in the fatty acid composition changes the fluidity of the membranes and, therefore, the ion transport across the thylakoid membrane. The desaturation of fatty acids also provides H^+^ ions, and hence implies that there is a reaction where H^+^ ions are needed [88].

The regulation of CAH3 in different CO_2_ conditions differs from the regulation of CAH1 or CAH2. It has been found that the activity and localization of CAH3 changes according to the CO_2_ conditions, unlike the case with CAH1 [85]. Blanco-Rivero et al. [85] discovered that the amount of mRNA or the actual protein did not increase significantly during acclimation to low CO_2_ conditions [85]. However, the activity of CAH3 increased due to phosphorylation, as did the amount of CAH3 in intrapyrenoid thylakoids at the expense of stromal thylakoids [85].

Additionally, the optimal pH of CAH3 is more acidic [87] than that of other CAs of *C. reinhardtii*. Benlloch et al. [87] measured the activity of CAH3 at different pH values, and discovered that the optimum was approximately pH 6.5 compared with that of the other CAs, which function best around a neutral pH. The activity also persists at a higher level than the activity of the other CAs at lower pH values [87].

A recent study showed that CAH3a associates with TAT2 and TAT3 proteins of the twin arginine translocation (Tat) pathway, and delivers substrate proteins to the thylakoid lumen [14]. The study also showed that the interaction between CAH3 and STT7 phosphorylates CAH3, CAH3 increases its catalytic activity when CO_2_ is low, and CAH3 converts HCO_3_^−^ to CO_2_ in thylakoid membranes that traverse the pyrenoid, supplying the pyrenoid with the high concentration of CO_2_ that is essential for CCM [14,82].

#### 2.1.4. β-Carbonic Anhydrase 4

Eriksson et al. reported the presence of a CA in *C. reinhardtii* that belongs to the β-CA family in 1995 [91]. The CAH4 is localized in the mitochondria of *C. reinhardtii*, and has a molecular mass of 20.7–22 kDa. The gene coding CAH4 is called *β-Ca1*, of which the whole nucleotide sequence has been examined, and was found to have 96% identity with another mitochondrial CA5 (CAH5) coding gene, *β-Ca2* [91]. *β-Ca1* is induced in low CO_2_ conditions, but not in high CO_2_ conditions; hence, it is present only when CO_2_ levels are high [91].

There have been many theories about the physiological role of CAH4 and CAH5. On one hand, Eriksson et al. [91] suggested that they are used in buffering reactions in changing CO_2_ conditions [91]. Glycine decarboxylation in photorespiration produces excessive amounts of CO_2_ and NH_3_ in low CO_2_ conditions. H^+^ is used because NH_3_ forms NH_4_^+^ at the pH of the mitochondrial matrix. Due to the need for H^+^, CAH4 catalyzes the hydration of CO_2_ to be faster in order to maintain the pH in the matrix [91]. On the other hand, Raven hypothesized that there might be a HCO_3_^−^ channel in the inner mitochondrial membrane; thus, both CAH4 and CAH5 have a role in preserving the CO_2_ [89].

There is also a third hypothesis for the function of CAH4 and CAH5, suggesting that they might provide HCO_3_^−^ for reactions catalyzed by phosphoenolpyruvate carboxylase where N is combined to C skeletons that can be later used in protein synthesis [89]. It has also been shown that because of this assumed function, the external NH_4_^+^ concentration is an essential regulator of the expression and function of CAH4 [89].

#### 2.1.5. β-Carbonic Anhydrase 5

Eriksson at al. identified CAH4 and CAH5 simultaneously in *C. reinhardtii* [91]. The two clones that code for CAH4 and CAH5 differ only slightly in their nucleotide sequences. In the coding area, the difference is only seven nucleotides, leading to one amino acid change at position 53, where serine is replaced by alanine [91]. In addition, the upstream regulating sites of *β-Ca1* and *β-Ca2* are very similar. Due to the striking similarity of *β-Ca1* and *β-Ca2,* the genes are likely to be duplicates that were selected because they increased the quantity of mtCA [90]. CAH4 and CAH5 lack any known functional difference, which also supports the gene-duplication assumption [90].

#### 2.1.6. β-Carbonic Anhydrase 6

Mirta et al. discovered CAH6 in 2004 [97], and showed it to be localized in the chloroplast stroma [97,98]. In contrast, localization studies performed by Mackinder et al. [14] recently showed that CAH6 is expressed in flagella and shows no detectable signal in chloroplasts [14]. To validate their findings, the authors analyzed the presence of CAH6 in proteomic datasets, and showed it in the flagellar proteome and in intraflagellar transport (IFT) cargo [14].

The cDNA of *Cah6* is 2886 bp long and encodes a 264-amino-acid-long polypeptide, CAH6. CAH6 has a calculated molecular mass of 26 kDa, but experimentally, it has a mass of 28.5 kDa in an SDS-polyacrylamide gel [97]. Some amount of CAH6 expression is induced in low CO_2_ conditions, but its expression levels are high in high CO_2_ conditions, similar to the case with many other CA isoenzymes in *C. reinhardtii*. CAH6 was believed to be involved in trapping CO_2_ that is leaking out of pyrenoids by converting it to HCO_3_^−^ and thus preventing C_i_ from leaving the chloroplast [97].

However, a recent study showing the localization of CAH6 to be in the flagella suggested that CAH6 is not required in the chloroplast, as its presence in the chloroplast may short circuit the CCM by converting CO_2_ from HCO_3_^−^ and its subsequent release away from Rubisco [14]. Indeed, this is the case at least in cyanobacteria, where the presence of CA disrupts the CCM [99]. *Chlamydomonas* are known to show chemotaxis toward HCO_3_^−^, and CAs have been implicated in C_i_ sensing, and hence may be directly involved in sensing C_i_ [14,100,101].

#### 2.1.7. β-Carbonic Anhydrase 7

Ynalvez et al. identified CAH7 in 2008 [92] by examining the sequences of two genes that code for CAs, namely, CAH7 and CAH8 [92]. The identified gene sequence of *Cah7* contains 5077 bp. The protein product of the gene *Cah7* has 399 amino acids, including 23 amino acids that are well conserved in β-CAs, as well as two cysteines and one histidine, which coordinate Zn^2+^. In addition, the researchers predicted that CAH7 has a transmembrane domain, and thus might be attached to a membrane [92].

The amount of CAH7 in the cell depends upon the levels of CO_2_ in the surroundings. The CAH7 is more abundant in low than in high CO_2_ conditions. Overall, CAH7 is expressed in lower amounts than most of the other CAs in *C. reinhardtii*. The location and physiological role of CAH7 in the cell is yet to be resolved [92].

#### 2.1.8. β-Carbonic Anhydrase 8

Ynalvez et al. identified *C. reinhardtii* CAH8 [92] in 2008 with CAH7, and both sequences were found to be closely related to each other [92]. The cDNA coding for CAH8 contains 2649 bp corresponding to a 333-amino-acid-long polypeptide. Furthermore, CAH8 has the same β-CA characteristics as CAH7, except that CAH8 has 22 of the 23 well-conserved amino acid residues. The molecular mass of CAH8 is approximately 40 kDa. Additionally, CAH8 has the same transmembrane domain near the C-terminus, which is similar to the transmembrane domain of CAH7. Immunolocalization studies have shown that CAH8 is located in the periplasmic space along with CAH1 and CAH2, but that the localization of CAH8 appears closer to the cell membrane compared with that of CAH1 [92].

The expression of CAH8 is constant in the algal cell, but the amount of enzyme is present in higher amounts in the presence of abundant CO_2_ than in the presence of lower CO_2_ amounts [92]. The overall expression of CAH8 resembles that of CAH6, as it is moderate among the CAs in *C. reinhardtii*. There are some theories regarding the function of CAH8. First, it has been suggested that, because CAH8 is closely related to the cell membrane, it would ensure the presence of CO_2_ near the membrane, despite the external pH conditions. Second, CAH8 has been proposed to be a part of the C_i_ delivery system as a carbon-binding protein. Third, an association with a pore or a channel has been proposed [92].

#### 2.1.9. β-Carbonic Anhydrase 9

The presence of CAH9 in *C. reinhardtii* was first reported in 2005 by Cardol et al. [71] from the genome sequencing project [71]. The RNA-Seq data that are available suggest that CAH9 is expressed at low levels (http://genomes.mcdb.ucla.edu/Cre454/) under the growth conditions that were used in the experiment at that time [12]. Since then, no further studies have been done on CAH9 expression and its role in *C. reinhardtii.*

#### 2.1.10. Limiting CO_2_ Inducible-B Protein/β-Carbonic Anhydrase Family

Limiting CO_2_ inducible-B protein (LCIB) is a key player in the eukaryotic algal CCM function in *Chlamydomonas reinhardtii* [100]. The LCIB gene encodes a novel chloroplast protein that consists of 448 amino acids with a predicted MW of 48 kDa, and forms a heteromultimeric complex with its close homolog LCIC; the complex may be tightly regulated or may require additional factors for proper functioning [14,98,100]. Interestingly, a recent study involving a double mutant analysis of LCIB/CAH3 showed that LCIB functions downstream of CAH3. It has been hypothesized that LCIB captures CO_2_ that leaked from the pyrenoid, possibly by unidirectionally hydrating CO_2_ back to HCO_3_**^−^ [101]**. Recently, in order to study the function of LCIB, a phylogenetically diverse set of recombinant LCIB homologs were produced in *E. coli* and purified [10]. Structural characterization of the purified proteins showed that three of the six homologs were structurally similar to the β-CAs at the level of the overall fold, zinc binding motif, and active site architecture. However, none of the three proteins showed CA enzymatic activity, and the lack of CA activity could be due to the widening of the intersubunit cleft, which affects active site integrity by causing disordering of the important His162/161 and Arg194/193 residues in the protein [10].

Based on the results of the study, it is proposed that LCIB in association with LCIC acts as a noncatalytic structural barrier for the leaked CO_2_ from the pyrenoid [10]. However, in order to elucidate the precise role of LCIB, further studies are needed involving the characterization of a LCIB–LCIC complex purified from a native source.

#### 2.1.11. γ-Carbonic Anhydrases

The gene *Glp1* that encodes γ-CAH1 was discovered in 2005 using the γ-CA protein sequence of *M. thermophila* and expressed sequence tag (EST) databases [70]. Similarly, the presence of three γ-CAs (CAG1, CAG2, and CAG3) in *C. reinhardtii* was also shown by two other groups, and these were predicted to be localized in the mitochondrial matrix [71,72].

The *Glp1* gene that codes for γ-CAH has seven exons and six introns, and encodes a putative protein of 312 amino acids [70]. The localization studies using prediction programs showed that this enzyme is localized in the cytoplasm or is secreted outside the cell. γ-CAH1 has approximately 40% similarity with the γ-CAH of *M. thermophila*, and has three histidine residues coordinating zinc at the active site of the enzyme. The recombinant proteins expressed in *E. coli* show no CA activity in either crude cell extracts or purified fusion protein [70]. However, the γ-CAHs may be active in the parent organism. 

**Figure 2:**
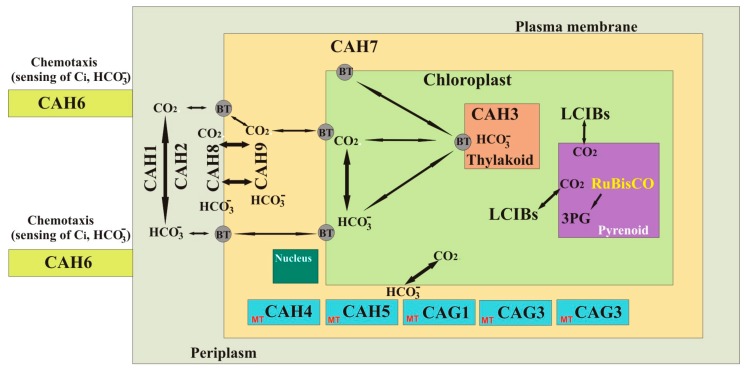
Schematic presentation of the *C. reinhardtii* model showing the roles of CAs in the cell and subcellular organelles. CAH1, CAH2, CAH3: α-Carbonic anhydrases; CAH4, CAH5, CAH6, CAH7, CAH8, and CAH9: β-Carbonic anhydrases; LCIB1, LICB2, and LCIB3: Low CO_2_-inducible proteins (β-CAs); CAG1, CAG2, and CAG3: γ-Carbonic anhydrases; BT: Bicarbonate transporters; *RuBisCO:* Ribulose-1,5-bisphosphate carboxylase oxygenase; 3PG: 3-phosphoglycerate. MT: mitochondria.

There are two additional γ-CAHs that have been annotated as subunits of the mitochondrial NADH dehydrogenase complex [70]. The sequence analysis showed that these γ-CAHs do not contain three histidine residues that are required for the catalytic activity of the CAs [70]. Based on the available studies, the γ-CAHs of *C. reinhardtii* are localized in the mitochondrial matrix, and are part of mitochondrial complex I. Interestingly, complex I of the mitochondrial electron transport chain (mETC) in *Arabidopsis thaliana* also contains three different protein domains that are homologous to γ-CAs [102]. Double mutants of *Arabidopsis thaliana* lacking γ-CAH1 and γ-CAH2 were analyzed for their role in development and physiology. The analysis of mutant strains of *A. thaliana* showed a developmental delay and an upregulation of complex II and complex IV, with increased oxygen consumption in mitochondrial respiration [102]. The results of this study suggest that the three γ-CAHs in *C. reinhardtii* may perform similar functions. The few studies on γ-CAHs were conducted a decade ago; therefore, the information on the physiological roles of these CAs is incomplete. We need more studies using bioinformatic and molecular tools for the structural and functional analysis of these γ-CAHs in order to know their precise roles in *C. reinhardtii*. Based on the latest information we propose a hypothetical model showing the localizations and functions of CAs in *C. reinhardtii* (Figure 2).

## 3. Conclusions and Future Directions

The CA enzymes belonging to different classes of CA gene families are found in vertebrates, invertebrates, plants, unicellular marine and fresh water algae, bacteria, and archaea. The CAs are localized in almost all of the tissues of higher animals and subcellular organelles of eukaryotic cells, and perform a variety of metabolic and physiological roles. In plants, several classes of CAs are found that are localized in subcellular organelles, are involved in CCM for photosynthesis, and perform other metabolic functions. Plant biologists have used marine and fresh water unicellular photosynthetic model organisms in order to study the precise metabolic roles of CA enzymes. The freshwater alga *C. reinhardtii* is one such model organism that has helped us to understand the metabolic and physiological roles of CAs mainly on CCM. However, the precise metabolic roles of most of the CA enzymes in this alga remain to be studied.

There has been a continuous interest in CA research in unicellular photosynthetic organisms, especially as the genomes of these algae have become available. The availability of bioinformatic and molecular tools has helped to study the precise metabolic roles of CAs in photosynthetic model organisms. In *C. reinhardtii*, researchers have attempted to study the localization and metabolic roles of three α-CAs. Contradictory reports have emerged on the precise localizations of the CAs, and only limited information is available on the physiological roles of six β-CAs and the newly reported LCIB protein family that belongs to the β-CA group. No studies are available on γ-CAs except the presence of three forms of this enzyme and their predicated localization in the mitochondrial matrix. The challenge for future researchers will be to determine the precise localization and biochemical roles of all 12 CAs and the newly discovered three LCIB family proteins.

It is important to identify the precise physiological roles for all of the CAs found in *C. reinhardtii*, which is an important model organism for studying fundamental processes such as photosynthesis. *C. reinhardtii* is the most commonly studied species of *Chlamydomonas* and has a relatively simple genome, which has been sequenced in many different strains, including the nonmotile strains. More importantly, various strains of *C. reinhardtii* have been developed for specific research purposes. In photosynthetic organisms, including *C. reinhardtii*, the role of CAs in CCM have been studied extensively. In addition to the fundamental roles of CAs in the reversible hydration of CO_2_, CAs have been shown to play important roles in defense mechanisms in plants and animals, protozoa, and bacteria. Therefore, it will be important to widen the perspective of CA studies in *C. reinhardtii* to cover not only pH regulation, but also other potential processes.

## Figures and Tables

**Table 1:** Details of the carbonic anhydrase (CA) gene family enzymes in living organisms.

CAs	Enzyme	Metal Ion	Organisms	Ref.
α	Monomeric, dimeric	Zn^2+^	Animals, prokaryotes, fungi, and plants	[8,32]
β	Multimeric	Zn^2+^	Plants, bacteria, and fungi	[8,32]
γ	Trimeric	Zn^2+^ or Fe, Co	Plants, archaea, fungi, and bacteria	[33,34]
ζ	Monomeric	Cd or Zn	Marine diatoms	[30,33,34]
δ	Monomeric	Co	Marine diatoms	[30,35,36]
η	Monomeric	Zn^2+^	*Plasmodium* spp.	[1,37]
θ	Monomeric	Zn^2+^	Marine diatoms	[4,9,10]

**Table 2:** Details of the 15 carbonic anhydrases found in *Chlamydomonas reinhardtii* belonging to the α, β, and γ gene families.

CA Protein	Chr	Gene Family	MW (kDa)	Location	Known/Predicted Physiological Roles of the CAs	References
CAH1 ^a^	4	α	78	Periplasm/late secretory pathway	Supply of Ci in low CO_2_	[66,73,74,75,76,77,78,79]
CAH2 ^a^	4		84	Periplasm/late secretory pathway	Supply of Ci in high CO_2_	[14,66,80,81]
CAH3 ^a^	9		29.5	Chloroplasts	Growth in low CO_2_	[14,82,83,84,85,86,87,88]
CAH4 *^,a^	5	β	21	Mitochondria	-	[14,89,90,91]
CAH5 *^,a^	5		21	Mitochondria	-	[14,40,41,42]
CAH6 ^a^	12		31	Flagella	CCM	[14]
CAH7 ^b^	13		35.79	Periplasm?	-	[92]
CAH8 ^a^	9		35.79	Periplasm	-	[92]
CAH9 ^a^	5		13.06	Cytosol	-	[14]
LCIB1 ^b^			48 ^c^	Chloroplasts	CO_2,_ uptake, CCM	[10]
LCIB2 ^b^			48 ^c^	Chloroplasts	CO_2,_ uptake, CCM	[10]
LCIB3 ^b^			48 ^c^	Chloroplasts	CO_2,_ uptake, CCM	[10]
CAG1 ^b^	9	γ	24.29	Mitochondria	Transport of mitochondrial CO_2_ to chloroplasts	[14,70,71,93]
CAG2 ^b^	6		31.17	Mitochondria	Transport of mitochondrial CO_2_ to chloroplasts	[14,71,72,93]
CAG3 ^b^	12		32.69	Mitochondria	Transport of mitochondrial CO_2_ to chloroplasts	[14,71,72,93]

* The amino acid sequences of these two β-CAs differ by a single amino acid. ^a^ CA activity is known; ^b^ CA activity is not known; ^c^ Predicted molecular weight. Chr = chromosome.
